# Early Detection of Neuroinflammation and White Matter Damage Following Dorsal Spinal Nerve Root Sectioning in a Nonhuman Primate Model

**DOI:** 10.1002/mrm.70213

**Published:** 2026-01-08

**Authors:** Feng Wang, John C. Gore, Li Min Chen

**Affiliations:** ^1^ Vanderbilt University Institute of Imaging Science Vanderbilt University Medical Center Nashville Tennessee USA; ^2^ Department of Radiology and Radiological Sciences Vanderbilt University Medical Center Nashville Tennessee USA; ^3^ Department of Biomedical Engineering Vanderbilt University Nashville Tennessee USA

**Keywords:** chemical exchange saturation transfer (CEST), diffusion tensor imaging (DTI), fiber damage, MRI, nerve root injury, quantitative magnetization transfer (qMT), relayed nuclear Overhauser enhancement (rNOE)

## Abstract

**Purpose:**

Dorsal rhizotomy, or spinal dorsal nerve root lesioning, is a surgical procedure used to treat intractable nerve pain by selectively severing sensory afferent nerve roots. This study aimed to evaluate whether multiparametric MRI, including diffusion tensor imaging (DTI), quantitative magnetization transfer (qMT), chemical exchange saturation transfer (CEST), and relayed nuclear Overhauser enhancement (rNOE), can sensitively detect structural and biochemical changes in the intact spinal cord following a focal dorsal nerve root section in a nonhuman primate model.

**Methods:**

In four squirrel monkeys, unilateral dorsal nerve roots at cervical segments C4 and C5 were surgically transected. MRI data were collected using a 9.4 T scanner with a custom saddle‐shaped transmit‐receive quadrature coil before and 1 week after lesioning. DTI‐derived fractional anisotropy (FA), axial diffusivity (AD), and radial diffusivity (RD); qMT‐derived pool size ratio (PSR); and CEST and rNOE effects extracted from five‐pool Lorentzian fitting of Z‐spectrum, were quantified across seven regions of interest.

**Results:**

At the lesioned dorsal nerve root bundles, FA, PSR, and rNOE (−1.6 ppm) values decreased, while RD and CEST (3.5 ppm) increased, consistent with fiber degeneration, demyelination, and inflammation. Similar, though less pronounced, changes were observed in the dorsal root entry zone, particularly within the first week post‐lesion.

**Conclusion:**

Multiparametric MRI enables region‐specific detection of spinal cord pathology, including axonal degeneration, demyelination, and possible neuroinflammation, as early as 1 week after dorsal nerve root injury. These results demonstrate its promise for noninvasive monitoring of post‐injury pathology and for evaluating therapeutic efficacy.

## 1 Introduction

Traumatic nerve root injuries typically occur in individuals who have experienced major trauma, such as high‐velocity accidents. Cervical injuries are most common due to the spine's mobility. These injuries can also arise from spinal dorsal sensory nerve root lesioning (dorsal rhizotomy), a neurosurgical procedure in which the sensory afferent nerve rootlets of the dorsal spinal roots are selectively severed to reduce pathological sensory inputs [1]. This procedure is used in children with cerebral palsy, individuals suffering from intractable cancer or neuropathic pain, and patients with spinal cord injury (SCI)‐related pain or spasticity [2]. Given the anatomical and physiological similarities between nonhuman primates (NHPs) and humans, NHPs serve as realistic and translationally relevant preclinical models for studying nerve root injuries and the resulting spinal cord pathology. In an NHP model of SCI, we previously demonstrated that resting‐state functional MRI revealed robust disruption in functional connectivity, while multiparametric MRI identified region‐specific alterations in microstructure and molecular composition within the injured spinal cord [3, 4]. These approaches offer detailed insights into underlying pathological processes. Unlike SCI, dorsal root sectioning occurs outside the spinal cord. However, the disruption of nerve bundles frequently leads to pathological changes within the spinal cord, which may contribute to or mediate the relief of clinical symptoms. In this study, we aimed to characterize those structural and biochemical changes using multiparametric MRI in an NHP model involving targeted unilateral dorsal root transection at two cervical spinal cord segments (C4 and C5). We hypothesized that focal, selective unilateral dorsal root sectioning would initiate afferent fiber degeneration, potentially leading to tissue damage extending into the central branches of the affected nerve root bundles and their entering zones—specifically, the dorsal root entry zone (DREZ).

Quantitative assessment of SCI remains challenging. Clinical diagnosis of spinal cord and peripheral nerve injuries primarily relies on anatomical MRI with varying contrasts [5, 6]. This limitation is partly due to technical constraints, such as the small size of the cord and nerves, local magnetic field inhomogeneity, relatively low signal‐to‐noise ratio (SNR), restricted scan time, and motion artifacts from cerebrospinal fluid (CSF) pulsations associated with cardiac and respiratory cycles [7, 8]. Currently, diffusion tensor imaging (DTI) is the most widely used quantitative MRI technique due to its relatively high reliability and straightforward data acquisition. DTI characterizes the anisotropy of water diffusion in white matter (WM) using a three‐dimensional tensor model, generating metrics that reflect microstructural features such as fiber density, orientation, and integrity [9, 10]. Advances in MRI hardware and imaging sequences have enabled DTI to be applied to various spinal pathologies, including traumatic injury [11, 12]. In addition, quantitative magnetization transfer (qMT) imaging can detect axonal demyelination and fiber loss, while also identifying abnormal changes in semisolid macromolecular content in tissue adjacent to injury sites [13, 14]. Chemical exchange saturation transfer (CEST) and relayed nuclear Overhauser enhancement (rNOE) provide sensitivity to molecular composition, offering additional information about lesion‐related metabolic and biochemical alterations, particularly in the early stages of injury [3, 15]. Together, CEST and rNOE provide a physiological window into cellular composition, metabolic state, and tissue acidosis.

In recent years, we have leveraged the capabilities of high‐field MRI to integrate DTI, qMT, CEST, and rNOE in a single session for evaluating tissue composition and biochemical changes associated with SCI in NHPs subjected to controlled dorsal column transection [3, 4, 13, 14, 15, 16, 17, 18]. We have also extended this approach to rodent models of lumbar contusion injury [19, 20, 21]. Multiparametric MRI has enabled comprehensive assessment of SCI progression and recovery and supported the identification of mechanism‐informed MRI biomarkers. This study applied our established multiparametric MRI approach to examine regional changes in the injured dorsal nerve root (DNR) bundles and the adjacent intact spinal cord following dorsal root transection in individual squirrel monkeys. The central aim was to investigate how damage to nerve root fibers located outside the spinal cord influences spinal cord integrity, particularly at the dorsal root‐spinal cord interface, where no direct trauma occurs. We also evaluated whether DTI, qMT, CEST, and rNOE can detect subtle pathological changes at the early stage (1 week) after nerve root sectioning. The insights gained from this study will help advance the use of multiparametric MRI in detecting and characterizing early and localized spinal cord pathology resulting from clinically relevant nerve root injuries.

## 2 Methods

### 2.1 Unilateral Dorsal Root Transection

Four adult male squirrel monkeys ( *Saimiri sciureus* , 6–8 years old) were studied. The cervical segments C4/5–C6/7 receive sensory input from the upper arm and hand. This injury model is designed to selectively and unilaterally disrupt DNRs of C4 and C5 segments. DNRs at the C4 and C5 segments were sectioned for squirrel monkeys. In brief, under surgical level anesthesia and aseptic conditions, the dorsal portion of the cervical spinal cord was exposed. Seven branches of DNRs were transected on one side before they enter the spinal cord using fine surgical scissors at both C4 and C5 levels. Each of the 7 nerve roots was separately cut and fully disconnected. The dura was replaced with a small piece of gelfilm, and the wound was enclosed. All procedures were approved by the Institutional Animal Care and Use Committee (IACUC) at Vanderbilt University and adhered to the NIH guidelines for the care and use of laboratory animals.

### 2.2 In Vivo MRI Data Acquisition

MRI data for each animal were acquired from the cervical spinal cord (C3 to C7) pre‐ and 1‐week post‐surgery to identify changes at the early stage. During MRI data acquisitions, each monkey was anesthetized with isoflurane (0.8%–1.2%), delivered in a 70:30 N_2_O/O_2_ mixture, and mechanically ventilated. Vital physiological signs were continuously monitored and maintained at stable levels throughout the imaging session.

**FIGURE 1:**
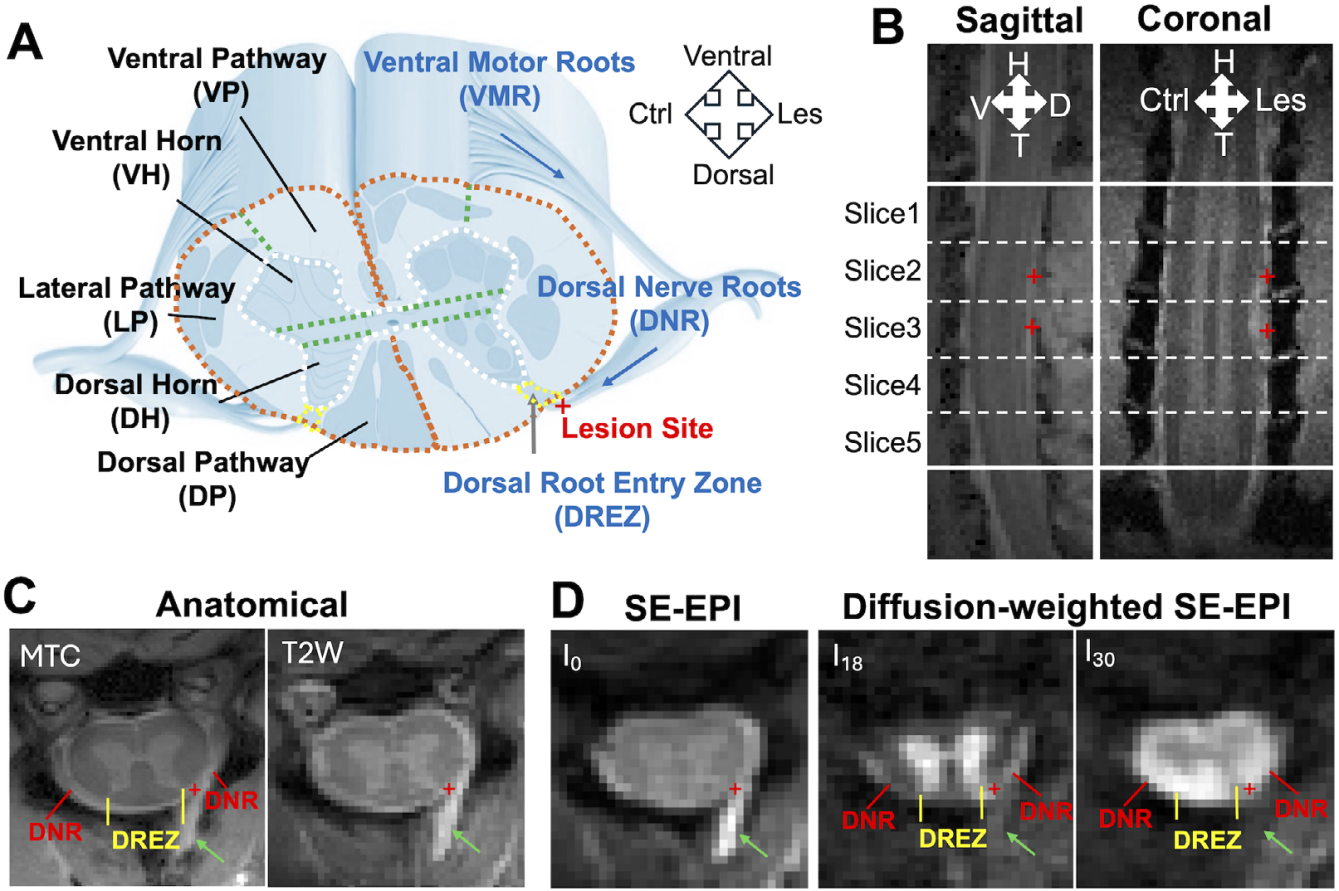
Anatomical images after unilateral dorsal nerve root lesion. (A) Schematic illustration showing the regions of spinal cord and lesion site (indicated by “+” at the dorsal nerve roots). White matter regions (VP, ventral pathway; LP, lateral pathway; DP, dorsal pathway) and gray matter regions (VH, ventral horn; DH, dorsal horn) are delineated by dashed lines. The dorsal root entry zones (DREZ) are highlighted by yellow dashed line. Ctrl, contralateral; Les, lesion. (B) MRI images with magnetization transfer contrast (MTC) in sagittal and coronal orientations. “+” symbols mark the targeted lesion sites at the C4 and C5 dorsal sensory nerve roots. H, head; T, tail. (C) High‐resolution anatomical MTC and T_2_‐weighted (T2W) images. (D) Spin‐echo echo‐planar image (SE‐EPI). I_0_ represents the non‐diffusion‐weighted SE‐EPI image, I_18_ and I_30_ correspond to the 18th and 30th diffusion‐weighted SE‐EPI images, with diffusion gradients more parallel and more perpendicular to the cord respectively. Green arrows indicate fluids accumulated after lesion. Red lines denote dorsal nerve root (DNR) bundles, and yellow lines indicate the DREZ. Images are from one representative subject 1‐week post‐lesion.

All MR images were acquired using a customized saddle‐shaped transmit‐receive quadrature surface coil (each of the two loops is 30 × 30 mm^2^, mounted on 45‐mm‐diameter cylindrical surface) positioned around the cervical spine region at 9.4 T. This coil was optimized to sample data from cervical spinal cord [22]. It reduced unwanted signals from distant moving tissues (such as moving chest) and increased SNR. Magnetization transfer contrast (MTC)‐weighted structural images were acquired in three orientations (axial, coronal, and sagittal) with enhanced contrast [3], facilitating the precise slice placements for DTI, qMT, and CEST data acquisition (Figure 1B).

The DTI data acquisition was centered around the lesion level C4–C5 (Figure 1). The diffusion‐weighted spin‐echo sequence used an echo planar imaging readout (TR/TE = 3000/33 ms, 4 shots, resolution = 0.333 × 0.333 mm^2^, slice thickness = 3 mm, 5 slices), with *b* values of 1000 s/mm^2^, sampling 30 directions (equally spaced). The qMT data were collected using a 2D MT‐weighted spoiled gradient echo sequence (TR 24 ms, flip angle = 7°, matrix size 128 × 128, 4 acquisitions, resolution = 0.25 × 0.25 × 3 mm^3^) for each spinal cord segment. Gaussian‐shaped saturation pulses (*θ*_sat_ = 220° and 820°, pulse width = 10 ms, 10 RF offsets with a constant logarithmic interval between 1 and 100 kHz) were used. The water saturation spectra (Z‐spectra) were acquired using a 5 s continuous wave (CW) saturation of amplitude 1.0 μT followed by a spin‐echo echo‐planar‐imaging acquisition (TR = 7.5 s, TE = 17.6 ms, 2 shots, matrix of 64 × 64, resolution = 0.5 × 0.5 × 3 mm^3^). RF offsets were from −2000 to 2000 Hz (−5 to 5 ppm at 9.4 T) with an interval of 80 Hz (0.2 ppm at 9.4 T). Reference scans were obtained at the beginning and the end of each acquisition using an RF offset at 100 kHz. High‐resolution MTC and T_2_‐weighted (T2W) anatomical images (Figure 1C) were acquired at 0.125 × 0.125 and 0.250 × 0.250 mm^2^ respectively, using the same geometry as the quantitative MRI. The total acquisition time for MRI data was approximately 1 h.

### 2.3 MRI Data Analyses

MRI data were analyzed using MATLAB (The MathWorks). Conventional DTI parameters, including fractional anisotropy (FA), axial diffusivity (AD), and radial diffusivity (RD), were quantified using the DTI Toolbox [23] based on diffusion data from the *b* = 1000 s/mm^2^ shell. The non‐diffusion‐weighted SE‐EPI (I_0_) images were diffeomorphically registered slice‐wise to the high‐resolution MTC images, and all other diffusion‐weighted volumes (I_1_–I_30_) were registered to I_0_ image using a rigid registration algorithm (Figure 1).

The qMT parameter, pool size ratio (PSR), was derived using the two‐pool Ramani model [13], which includes free water and bound water pools. The amplitudes of CEST and rNOE effects were quantified from Z‐spectra. The contribution of the semisolid magnetization transfer (MT) effects to the Z‐spectrum acquired at a saturation power of 1.0 μT was approximated as a constant due to its broad and slowly varying profile across the RF offset range [16, 24, 25]. The processing algorithm first inverted the Z‐spectrum between −5 and 5 ppm and removed the residual baseline caused by semisolid MT effects, setting the signal at 5 ppm to zero [16, 24, 25]. A nonlinear optimization routine was then applied to decompose the baseline‐corrected spectrum into its constituent components. Peak amplitudes corresponding to CEST effects at 3.5 and 2.0 ppm, and rNOE effects at −1.6 and −3.5 ppm, were extracted using a five‐pool Lorentzian fitting model (RF offsets: 3.5, 2.0, 0, −1.6, and −3.5 ppm).

High‐resolution MTC, T2W and diffusion‐weighted images were used as references for the manual selection of regions of interest (ROIs) for quantification, based on the known anatomy and landmarks in the monkey [26]. Seven ROIs were manually delineated along the regional boundaries using MATLAB, including WM in lateral pathway (LP), ventral pathway (VP), dorsal pathway (DP), DREZ, and DNR, and gray matter (GM) in ventral horn (VH) and dorsal horn (DH).

### 2.4 Statistical Analysis

A two‐sided Wilcoxon rank sum test was performed to obtain *p* value and evaluate the statistical significance of differences in affected C4 and C5 segments across subjects (number of injured segments = 8) for each individual ROI, comparing the lesion and contralateral non‐lesioned side with pre‐lesion healthy tissues. A *p*‐value < 0.05 was considered statistically significant.

## 3 Results

### 3.1 Nerve Root Injury Visible on Anatomical MTC and Diffusion‐Weighted Images

One week after unilateral dorsal root nerve sectioning, MTC and T2W images clearly revealed the injury levels and affected spinal cord segments (Figure 1). The seven nerve bundles at two cervical levels were transected before entering the spinal cord at near‐horizontal angles (Figure 1A). As a result, hyperintensities appeared along the dorsal surface of the spinal cord in both sagittal and coronal MTC images (Figure 1B). In axial MTC and T2W images, the accumulated fluids appeared hyperintense (green arrows in Figure 1C). The cervical nerve root bundles were well visualized in the high‐resolution MTC image (Figure 1). In diffusion‐weighted images, fluid signals were much darker than in non‐diffusion‐weighted images (green arrows in Figure 1D), which enhanced the visibility of DNR bundles. The DNR bundles became more visible when the surrounding CSF and accumulated fluid appeared darker in the selected 18th diffusion‐weighted SE‐EPI image I_18_ (Figure 1D), enhancing the contrast between WM in different orientation. On the lesion side, the DREZ and lesion site showed signal intensity changes in diffusion‐weighted images, appearing either hypointense (in the 30th diffusion‐weighted image I_30_) or hyperintense (in I_18_), depending on whether the diffusion gradient direction was parallel or perpendicular to the spinal cord (Figure 1D). These observations highlight how specific diffusion‐weighted contrasts can help differentiate WM fibers in different orientations. In summary, tissue abnormalities in the DNR and DREZ regions were clearly detected and distinguished using a combination of MTC and diffusion‐weighted images.

### 3.2 Multiparametric MRI Reveals Region‐Specific Changes in the Spinal Cord

**FIGURE 2:**
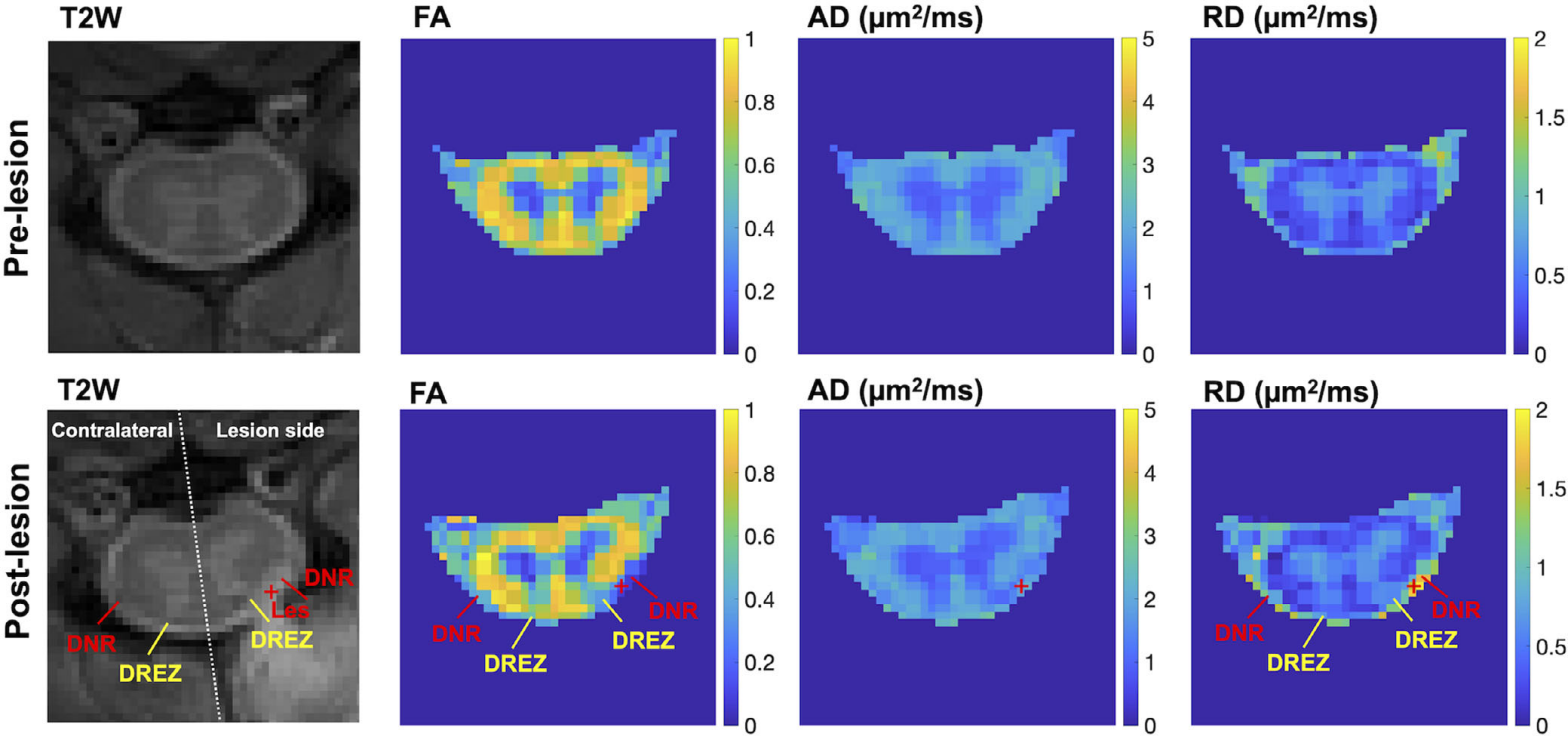
Comparison of diffusion tensor imaging (DTI)‐derived parametric maps in the selected spinal segment in pre‐lesion versus post‐lesion conditions. AD, axial diffusivity; FA, fractional anisotropy; RD, radial diffusivity. The red cross marks the lesion site at the dorsal sensory nerve roots. DREZ, dorsal root entry zone; DNR, dorsal nerve roots; Les, lesion site, indicated by the “+” symbol. Unilateral changes in DNR and DREZ regions are detected and indicated in FA and RD maps acquired post‐lesion.

**FIGURE 3:**
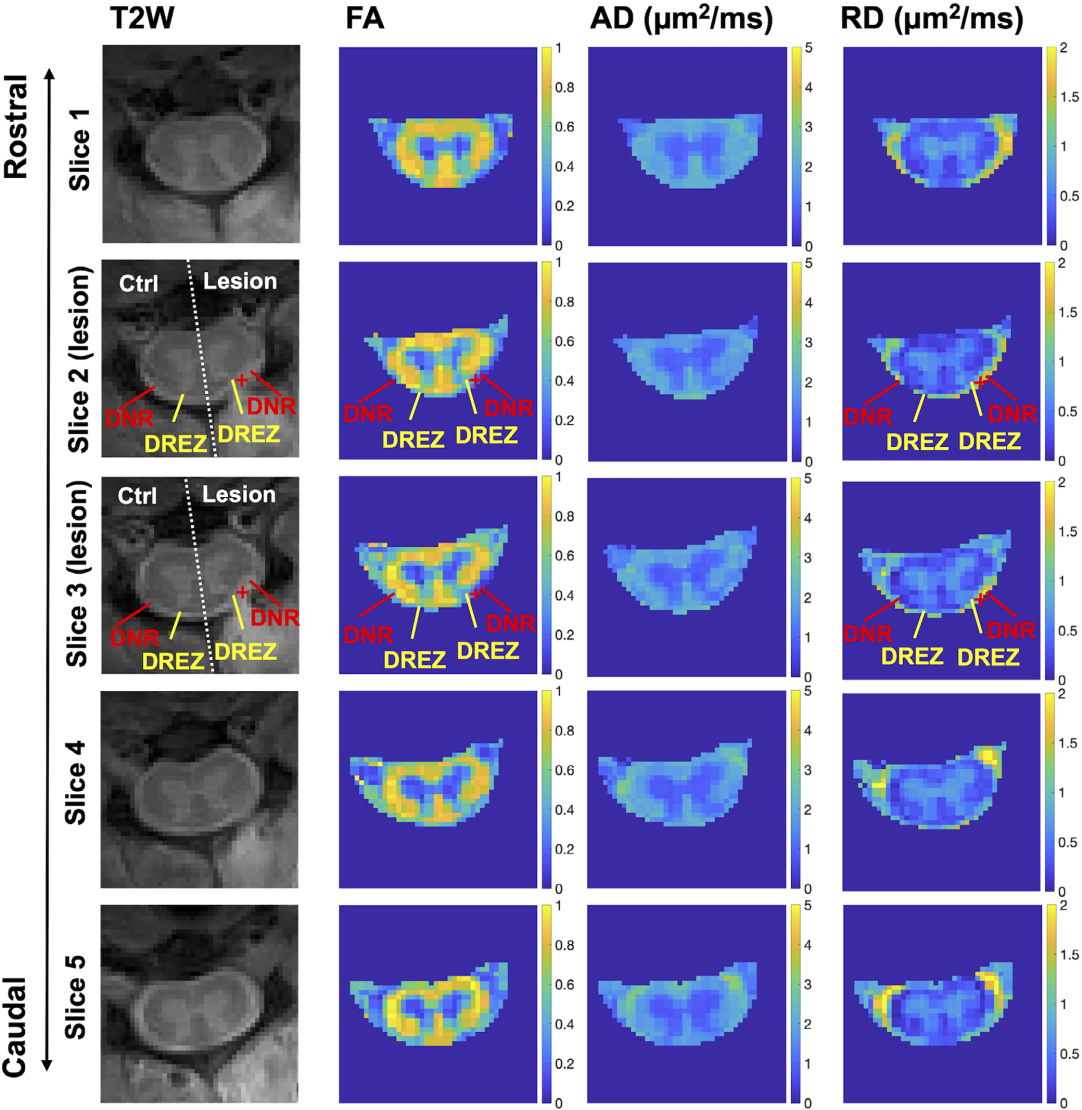
Comparison of DTI‐derived parametric maps across spinal cord segments post‐lesion. AD, axial diffusivity; FA, fractional anisotropy; RD, radial diffusivity. DNR, dorsal nerve roots; DREZ, dorsal root entry zone. The lesion site is indicated by the “+” symbol. Ctrl, contralateral side of spinal cord. Unilateral changes in DNR and DREZ regions on the lesion side are evident in FA and RD maps acquired post‐lesion.

Diffusion parametric maps revealed structural changes within the spinal cord, even though the nerve root injury occurred outside the cord. The lesion site in the DNR region (marked by a red cross in Figure 2) exhibited drastic increased RD and decreased FA. The adjacent DREZ inside the spinal cord also showed decreased FA and increased RD (yellow arrow in Figure 2), compared to the contralateral side. Notably, no substantial changes were detected in other ipsilateral regions such as the dorsal horn, dorsal pathway, or lateral pathway. These unilateral changes in diffusion measures were consistently observed at the two spinal segments that underwent nerve root sectioning (slices 2 and 3) when comparing across slices (Figure 3).

**FIGURE 4:**
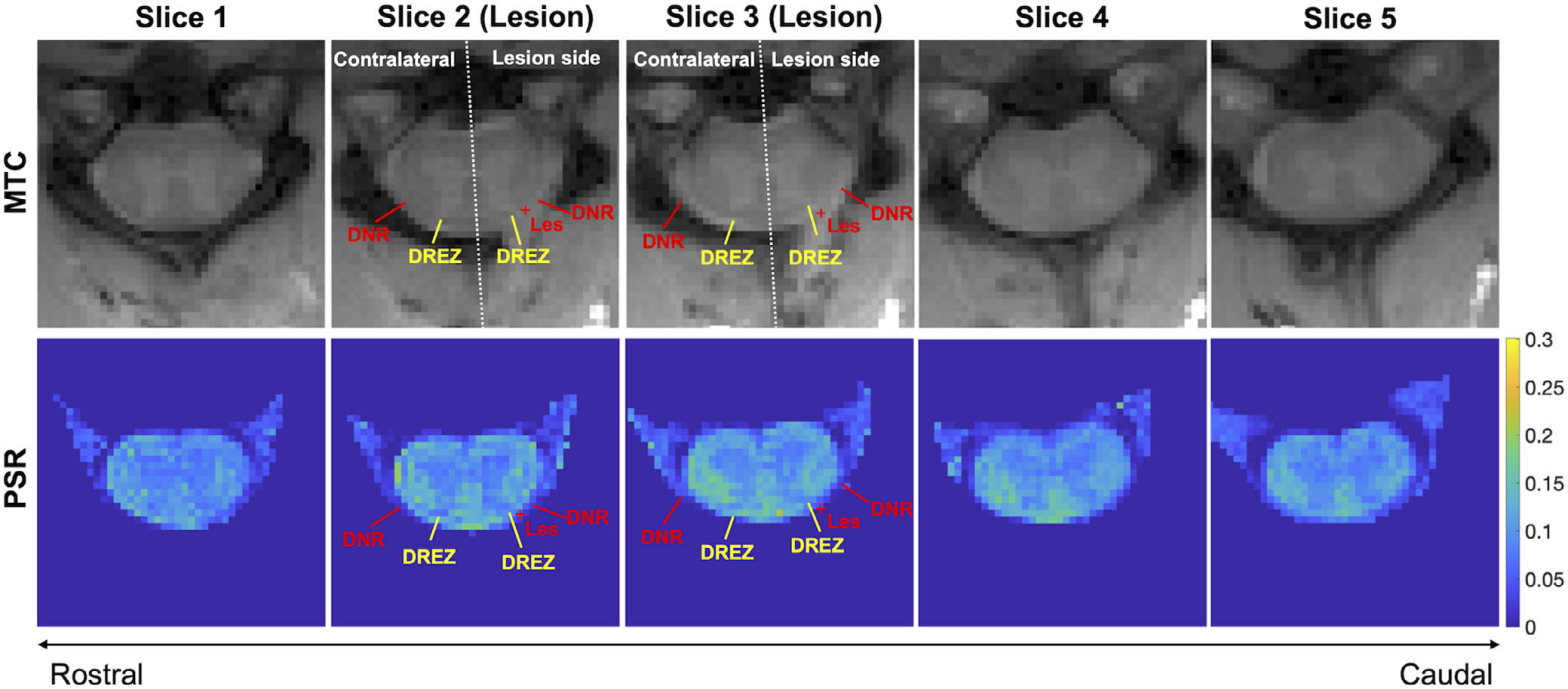
Comparison of pool size ratio (PSR) maps from quantitative magnetization transfer MRI. DNR, dorsal nerve roots; DREZ, dorsal root entry zone; Les, Lesion site; MTC, magnetization transfer contrast, indicated by the red “+” symbol. Unilateral changes in DNR and DREZ regions are evident in PSR maps. Results are from a representative subject following dorsal nerve root section.

PSR maps, derived from qMT, also revealed changes at and near the lesion sites at the two segments underwent sectioning on the lesion side 1‐week post‐injury. At 1‐week post‐injury, the DNR on the lesion side showed reduced PSR values compared to the contralateral side (Figure 4). A slight PSR decrease was also observed in the ipsilateral DREZ.

**FIGURE 5:**
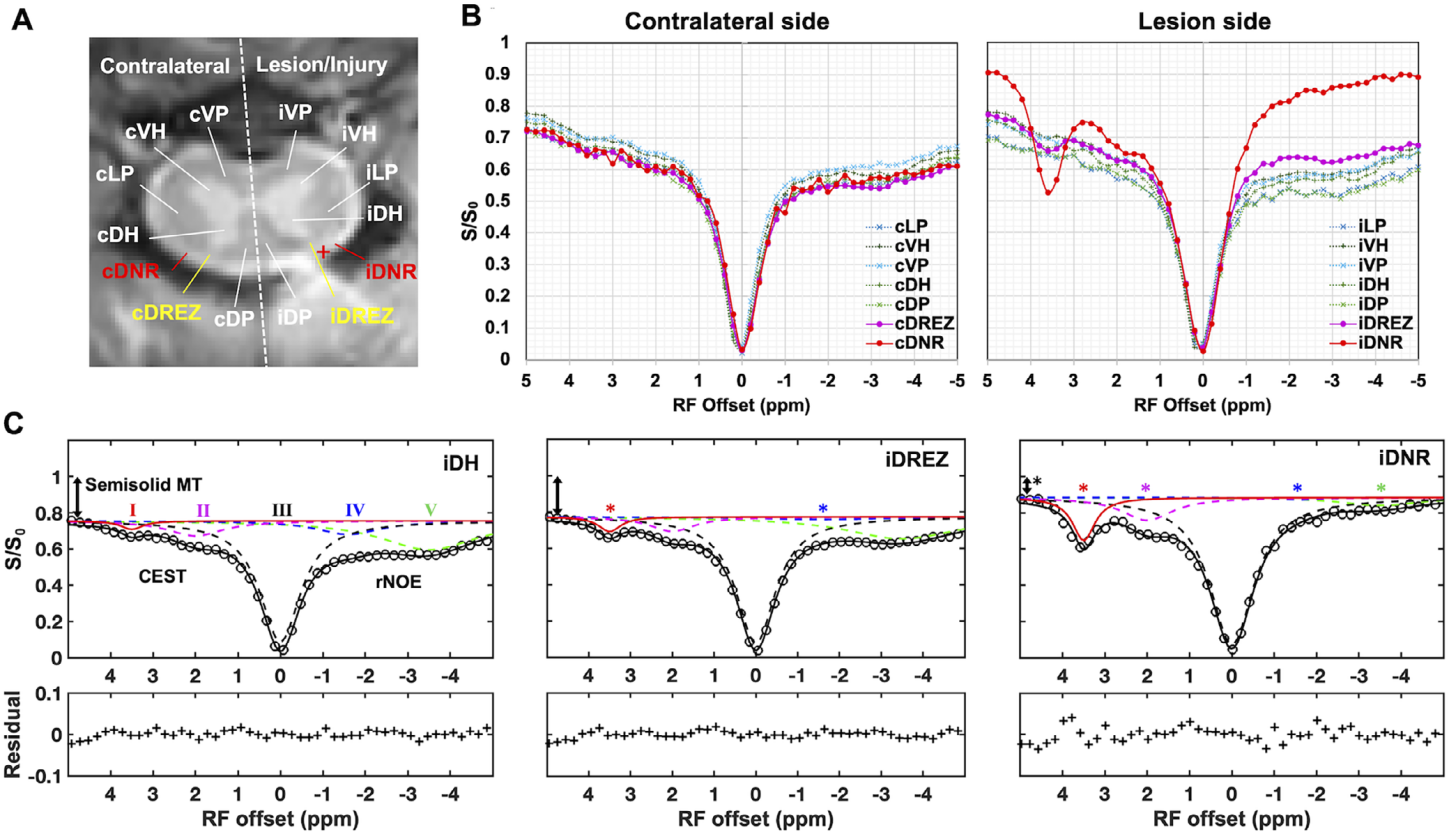
Comparison of averaged regional Z‐spectra. (A) Regions of interest. White matter regions include VP (ventral pathway), LP (lateral pathway), and DP (dorsal pathway). Gray matter regions include VH (ventral horn) and DH (dorsal horn). DREZ (dorsal root entry zone); DNR, dorsal nerve roots. The letters “c” and “i” in the ROI labels indicate contralateral and injury side, respectively. The red cross marks the lesion site of DNR. (B) Comparison of the regional spectra on the contralateral and lesion sides in spinal cord segments that underwent dorsal nerve root injury. (C) Selected ROIs showing results from 5‐pool Lorentzian fitting (3.5, 2.0, 0, −1.6, −3.5 ppm RF offsets). CEST, chemical exchange saturation transfer; rNOE, relayed nuclear Overhauser enhancement. Pool I, CEST (3.5); Pool II, CEST (2.0); Pool III, direct saturation on free water, DS (0.0); Pool IV, rNOE (−1.6); Pool V, rNOE (−3.5). Double arrows indicate semisolid magnetization transfer (MT) effects. Asterisks denote significant changes in the amplitude of the respective fitted peak. Results are from a representative subject after lesion.

The Z‐spectra of seven ROIs on contralateral and lesion sides were compared (Figure 5B). Peak amplitudes at different RF offsets were derived from 5‐pool fitting of each Z‐spectrum to assess CEST and rNOE effects (Figure 5C). The injured DNR (iDNR) and DREZ (iDREZ) showed increased CEST effects around 3.5 ppm RF offset and decreased rNOE at −1.6 ppm RF offset (red and blue asterisks, Figure 5C). The iDNR region also showed a reduced semisolid MT effect at 5 ppm RF offset (double‐headed arrow) and diminished rNOE effect at −3.5 ppm RF offset (green asterisk), likely due to severe nerve root damage and partial volume effects from fluid accumulation. Other nearby regions, such as the ipsilateral dorsal horn (iDH), showed no evident abnormalities 1‐week post‐injury (Figure 5B,C).

### 3.3 Group‐Level Comparison of DTI, qMT, and CEST Metrics Across ROIs

**FIGURE 6:**
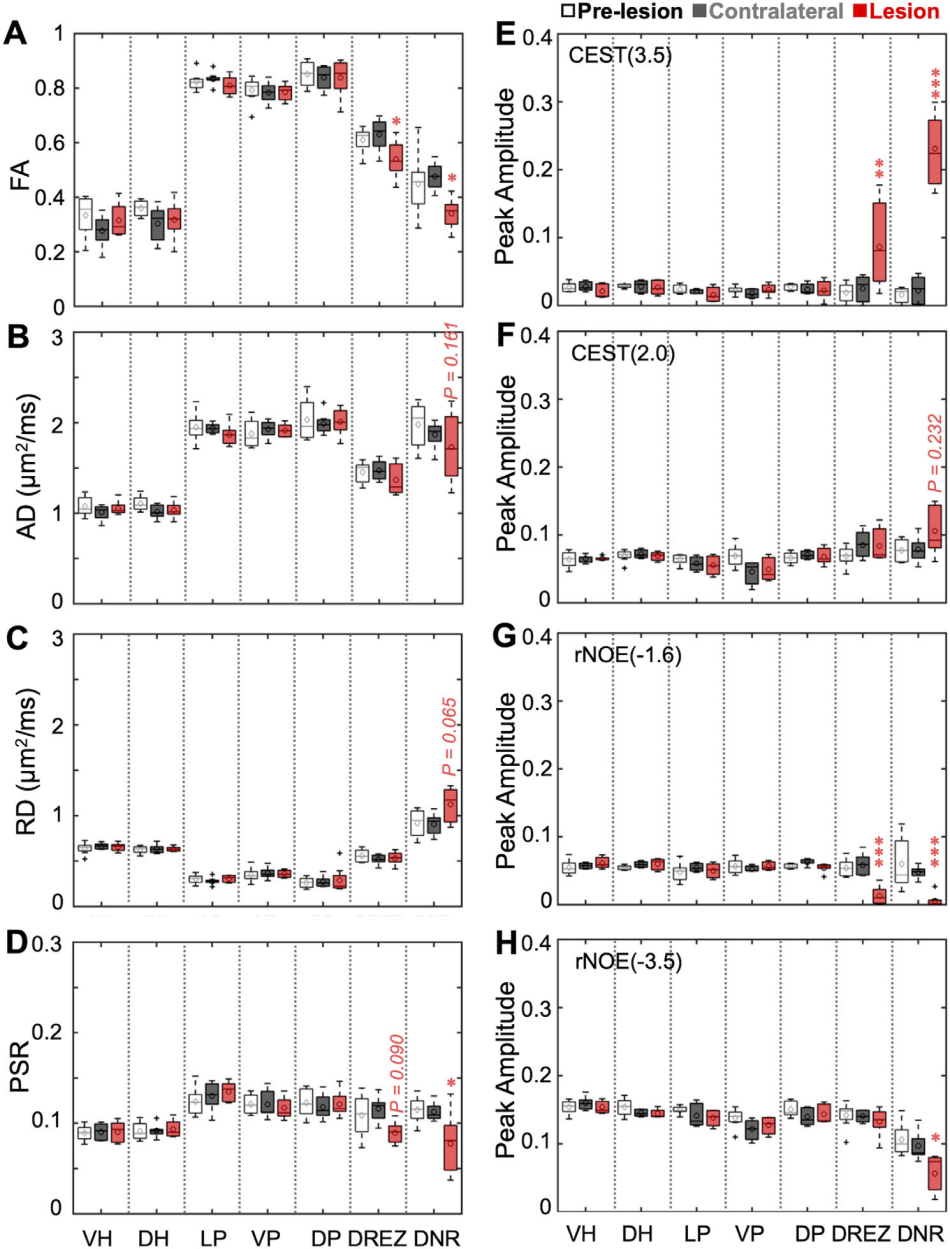
Group‐level comparison of multiparametric MRI metrics across specific ROIs in spinal cord segments (C4 and C5) that underwent nerve root injury. Boxplots of MRI measures from the non‐lesioned contralateral side and lesion side post‐lesion, along with values from pre‐lesion healthy spinal cord segments (*n* = 8). ROIs include three white matter regions (VP: ventral pathway, LP: lateral pathway, DP: dorsal pathway); two gray matter regions (VH: ventral horn, DH: dorsal horn); and two additional regions DREZ (dorsal root entry zone), and DNR (dorsal nerve roots). Definitions of these ROIs are illustrated in Figures 1 and 5. (A–C) Diffusion metrics FA (fractional anisotropy), AD (axial diffusivity), and RD (radial diffusivity). (D) PSR (pool size ratio) from quantitative magnetization transfer. (E–H) Peak amplitudes showing the CEST and rNOE effects at different RF offsets, including CEST (3.5), CEST (2.0), rNOE (−1.6), and rNOE (−3.5), extracted from Z‐spectra. In the boxplots, middle lines indicate medians and markers indicate mean values. *p < 0.05, **p < 0.01, and ***p < 0.001 versus the corresponding regional values in normal spinal cords (*Wilcoxon rank sum test*).

We performed group‐level, ROI‐based analyses of the MRI measures across seven ROIs (VH, DH, LP, VP, DP, DREZ, and DNR) in pre‐lesion and injured spinal cords (Figure 6). Significant changes in the lesioned DNR were detected in FA (Figure 6A), PSR (Figure 6D), CEST at 3.5 ppm (Figure 6E), and rNOE at −1.6 and −3.5 ppm (Figure 6G,H). Although AD decreased and RD increased in the lesioned DNR (Figure 6B,C), these changes were not statistically significant (*p* = 0.161 and *p* = 0.065, respectively). Similarly, the increase in CEST at 2.0 ppm was not significant (*p* = 0.232). The DREZ on the lesion side exhibited significant decreases in FA and rNOE (−1.6) and an increase in CEST at 3.5 ppm. PSR decreased by approximately 35.4% in the injured DNR and by 13.1% in the adjacent DREZ, indicating alterations in semisolid MT effects associated with immobile macromolecules. In contrast, the rNOE at −1.6 ppm decreased by 85.8% in the DNR and 72.5% in the DREZ, substantially greater than the corresponding PSR reductions, suggesting pronounced changes in mobile macromolecular components, such as proteins and lipids. None of the other ROIs, including those adjacent to the lesion (e.g., VH, VP, LP, VP, and DP), exhibited significant changes at this early post‐lesion time point.

### 3.4 Regional Correlations Among MRI Measures

**FIGURE 7:**
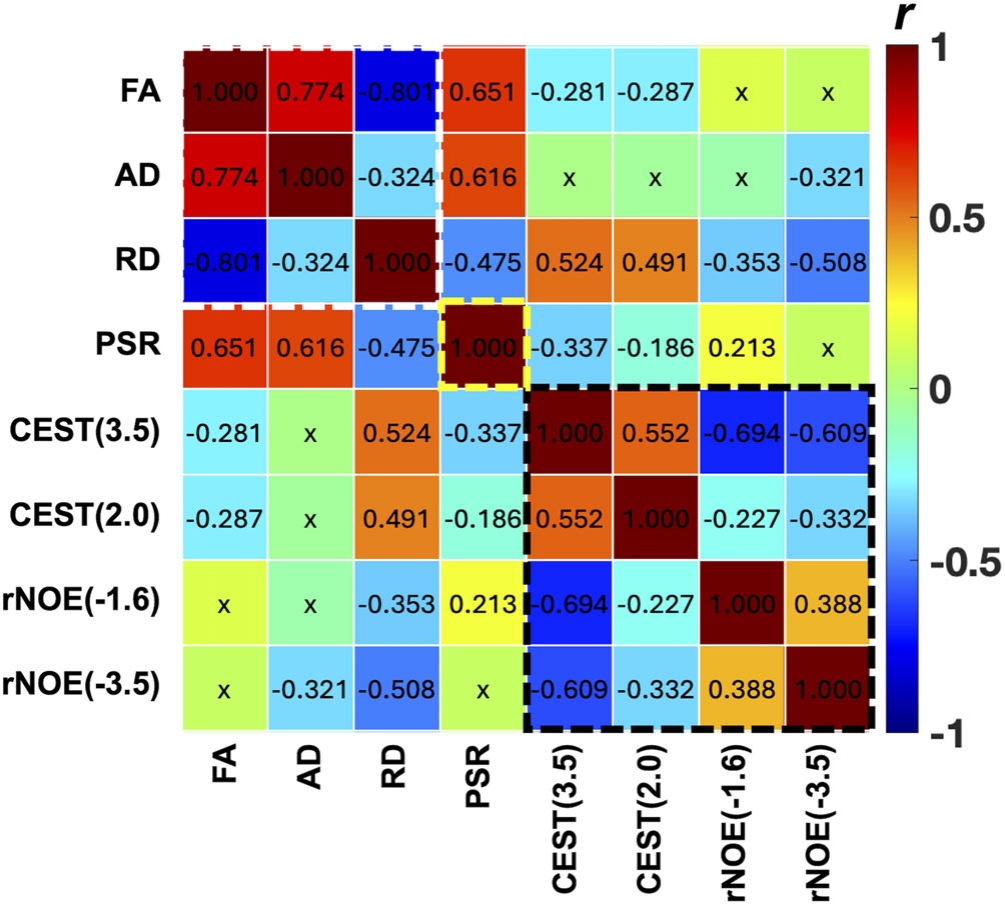
Regional correlations among MRI measures. Correlation coefficients (*r* values) are shown. “x” indicates correlation that is not significant (*p* > 0.05). Diffusion measures: FA, fractional anisotropy; AD, axial diffusivity; RD, radial diffusivity. PSR, pool size ratio from quantitative magnetization transfer. CEST (3.5), CEST (2.0), rNOE (−1.6), and rNOE (−3.5) are peak amplitudes at RF offsets 3.5, 2.0, −1.6, and −3.5 ppm, respectively, derived from Z‐spectral fitting. A total of 168 entries per metric were included in the correlation analysis.

To investigate the relationships among MRI metrics, we performed correlation analyses across ROIs. Figure 7 shows the correlation matrix of all paired comparisons using values from the seven ROIs across the two lesioned segments in four subjects, before and after lesioning (*n* = 168). Strong correlations (|*r*| > 0.6) were observed between metrics from the same MRI modality, such as positive correlations between FA and AD, and negative correlations between FA and RD. Additionally, CEST at 3.5 ppm was inversely correlated with rNOE effects. In addition, PSR from qMT also exhibited strong positive correlations with DTI‐derived FA and AD.

## 4 Discussion

Multiparametric MRI revealed complex, region‐specific pathological changes in the lesioned DNR and adjacent spinal cord tissue following nerve root injury. These changes likely reflect a combination of inflammation, demyelination, and axonal degeneration in the regions surrounding the lesion site.

### 4.1 Region‐Specific Tissue Changes Induced by Dorsal Rhizotomy

Following nerve injury, inflammation, demyelination, and axonal degeneration occur in a sequential and interconnected manner [27]. Inflammation is initiated by the primary injury, leading to immune cell activation that contributes to demyelination through breaking down of the myelin sheath. This loss of myelin, combined with direct axonal damage, results in distal axonal degeneration [27]. The resulting tissue property changes are region‐specific. This study aimed to characterize these pathological processes at both the injury site—DNR and the distant, non‐injured DREZ within the spinal cord, revealing distinct region‐specific alterations.

In the injured DNR region (Figure 6E–H), increased CEST signals at 3.5 and 2.0 ppm were accompanied by reduced rNOE effects at −1.6 and −3.5 ppm. The elevated CEST signal at 3.5 ppm likely reflects local increases in mobile proteins and peptides, as well as protein degradation during inflammation [27, 28]. The signal increase at 2.0 ppm may indicate elevated levels of creatine and amino acids, along with more exposed arginine side chains (guanidinium protons) in mobile proteins and peptides [24, 29, 30, 31, 32]. These changes are associated with neurotransmitters or metabolites involved in altered neuronal activity and inflammatory responses [33, 34]. The rNOE effect at −1.6 ppm, which is sensitive to aliphatic protons in lipids and membrane proteins, diminishes in response to tissue damage. The opposing changes in CEST (3.5) and rNOE (−1.6) in the injured DNR are consistent with protein degradation and protein denaturation [35, 36]. Moreover, decreased FA and AD further support the presence of fiber degeneration. Concurrent reductions in AD and increases in RD are consistent with both demyelination and axonal degeneration. Additionally, PSR from qMT, which is sensitive to myelin content, also decreased in the DNR, reinforcing evidence for demyelination. Collectively, these changes suggest a disrupted local chemical environment indicative of axonal degeneration, demyelination, and inflammation in the DNR region.

In the adjacent DREZ, the pattern of increased CEST (3.5), decreased rNOE (−1.6), and no significant changes in CEST (2.0) likely reflects protein degradation or unfolding [35, 36]. Although AD and RD did not show significant changes, a marked decrease in FA suggests disruption of local microstructure, including fiber organization and orientation. The absence of significant AD changes suggests that axonal degeneration is minimal at this stage. A slight reduction in PSR (*p* = 0.090), without corresponding RD alterations, points to mild demyelination. Together, these changes indicate disrupted fiber organization, protein denaturation, and mild demyelination in the adjacent DREZ.

These findings provide compelling in vivo evidence that focal unilateral dorsal root sectioning initiates afferent fiber degeneration. This degeneration subsequently gives rise to pathological alterations that extend into the central branches of the injured nerve root bundles and their spinal cord entry zones.

### 4.2 Advantages of Multiparametric MRI for Detecting Early‐Stage Changes After Nerve Root Sectioning

Our results demonstrate that multiparametric MRI provides a sensitive, noninvasive approach for detecting spinal cord pathology associated with dorsal root injury. The acquisition and comparison of multiparametric MRI maps enables differentiation among potential overlapping pathologies such as demyelination, axonal degeneration, and inflammation.

Reduction in the diffusion measure FA provides general insights into WM microstructural integrity, reflecting both demyelination and axonal degeneration. AD offers additional specificity: decreased AD suggests axonal injury, whereas increased RD can indicate both axonal damage and myelin loss [37]. Axonal degeneration disrupts the directional coherence of WM tracts, leading to decreases in both FA and AD. Under normal conditions, the myelin sheath restricts water diffusion in the radial direction; consequently, demyelination results in elevated RD due to the loss of this barrier. PSR, derived from qMT, reflects macromolecular content and is particularly sensitive to changes in myelin [38]. In our experimental model, dorsal root injury induced detectable alterations in both the central branches of dorsal nerves and the adjacent DREZ. In the injured DNR, reductions in FA and AD suggest axonal degeneration, while decreased PSR and increased RD indicate demyelination. In the adjacent DREZ, FA significantly decreased while AD remained unchanged, suggesting disruption in fiber organization without overt axonal loss. A modest reduction in PSR, accompanied by stable RD values, implies mild demyelination at the nerve entry zone.

CEST imaging relies on the chemical exchange of protons between specific biomolecules (e.g., proteins, metabolites) and bulk water, providing insight into tissue pH and the concentration of mobile proteins, peptides, and metabolites with exchangeable protons [39, 40]. CEST signals at 3.5 ppm RF offset reflect amide proton transfer effects and can be used to assess protein degradation, denaturation, and tissue acidosis [41, 42]. Signals at 2.0 ppm arise from amine‐water proton exchange and provide information about cellular activity through changes in amino acid and metabolite levels, such as arginine and creatine [29, 30]. The rNOE signals originate from the transfer of nuclear spin polarization via cross‐relaxation and are sensitive to mobile macromolecules containing aliphatic protons, including proteins, carbohydrates, and membrane lipids [36]. Protein degradation or denaturation leads to reductions in rNOE signals [35, 36]. Inflammatory processes introduce cellular infiltration and the release of biochemical mediators, altering tissue pH, composition, and cellular activity [27]. These changes may manifest in CEST and rNOE signals. The molecular profile varies depending on the specific byproducts released from damaged axons and surrounding structures [16]. In the injured DNR, elevated CEST signals at 3.5 and 2.0 ppm likely reflect changes in tissue composition associated with cellular activity and inflammation [27]. Concurrent increases in CEST at 3.5 ppm and decreases in rNOE at −1.6 ppm further suggest protein degradation and denaturation [35, 36]. In the adjacent DREZ, the most pronounced changes were observed in CEST (3.5) and rNOE (−1.6), which shifted in opposite directions, consistent with protein degradation and denaturation [35, 36].

### 4.3 Interpretation of Correlations Among MRI Metrics

Strong correlations were observed between metrics from the same imaging modality (Figure 7), suggesting that these parameters covary and provide complementary insights into different aspects of spinal cord pathology. The high correlation between amide and amine CEST pools implies a shared origin in mobile proteins and peptides under normal physiological conditions. Following injury, degradation and denaturation of macromolecules, along with the release of metabolites, small peptides, and amino acids, likely contribute to the observed negative correlations between rNOE and CEST metrics.

Among DTI and qMT metrics, RD showed the strongest correlation with CEST (3.5), indicating that RD increases are closely associated with overall tissue damage (Figure 7), including both axonal degeneration and demyelination. AD did not show significant correlations with CEST metrics, as reductions in AD are primarily linked to axonal injury. Post‐lesion decreases in PSR and FA were consistent with WM fiber damage, and these two measures exhibited a strong positive correlation. This relationship reinforces their combined utility as markers of subtle WM abnormalities within the intact spinal cord. PSR correlated slightly more strongly with CEST (3.5) than FA, possibly because PSR directly reflects the fraction of intact immobile macromolecular pools, whereas CEST (3.5) highlights biochemical changes associated with macromolecular degradation. FA, while significantly correlated with PSR due to the organized fiber structure of the spinal cord, does not directly measure macromolecular content and may be less relevant in regions with disrupted fiber architecture. Despite these distinctions, both PSR and FA reliably decreased in regions of fiber loss and exhibited strong correlation with each other, supporting their potential as imaging biomarkers for nerve injury and demyelination.

### 4.4 Challenges and Opportunities in Multiparametric MRI of Dorsal Root Injuries

In this study, we present MRI‐based evidence that transection of DNRs prior to their entry into the spinal cord induces measurable changes in WM microstructure and tissue composition within the intact spinal cord as early as 1‐week post‐injury. This finding is clinically relevant, as selective dorsal rhizotomy is a neurosurgical procedure used to alleviate pathological sensory input in conditions such as cerebral palsy, neuropathic pain, and SCI‐related spasticity [2].

The integration of multiparametric MRI metrics enables sensitive detection of subtle, region‐specific spinal cord pathology shortly after dorsal root injury. By examining coordinated changes in diffusion and molecular imaging parameters, it is possible to differentiate among demyelination, axonal degeneration, and inflammation, thereby enhancing our understanding of injury progression. The ability to noninvasively monitor delicate regional alterations is critical for both preclinical and clinical studies, especially when investigating secondary effects arising from upstream dorsal sensory disruptions. Detecting these pathological changes at the central terminals of sparsely distributed nerve roots is especially challenging, as they may span one to two spinal segments rostrally and caudally. The sizes of cervical spinal nerve root bundles vary along the spinal cord and differ between the dorsal and ventral sides in both humans and NHPs. Post‐mortem studies in humans have shown that DNR bundles are generally larger than ventral nerve root (VNR) bundles. Across cervical levels C1 to C8, the average diameters are 3.42 ± 0.74 mm (mean ± SD) for DNRs and 1.74 ± 0.56 mm for VNRs [43]. In squirrel monkeys, in vivo structural MR images (in coronal and sagittal orientations) show average diameters of approximately 1.56 ± 0.29 mm for DNRs and 0.76 ± 0.15 mm for VNRs across comparable cervical levels. Post‐mortem measurements indicate a DNR diameter of 1.32 ± 0.19 mm across C2 and C7, with an estimated tissue shrinkage of 15.8%. Based on post‐mortem measures, the DNR bundles in squirrel monkeys are approximately 38.5% the size of those in humans. Despite these differences in absolute size, both species exhibit similar anatomical characteristics, including segmental size variation and consistently larger DNRs compared to VNRs. For context, the cervical spinal cord itself averages approximately 8 × 12 mm^2^ in humans and 4 × 6 mm^2^ in squirrel monkeys, underscoring the overall proportional scaling of neural structures across species. In this context, multiparametric MRI offers a powerful tool to monitor structural and neurochemical changes and interpret their functional implications following nerve root injury.

## 5 Conclusions

This study demonstrates that multiparametric MRI can detect localized structural and biochemical changes within the intact spinal cord following unilateral dorsal root nerve section in a nonhuman primate model of dorsal rhizotomy. One‐week post‐injury, MRI revealed early signs of WM fiber degeneration, demyelination, and neuroinflammation at the central terminals of the dorsal root, as well as fiber disorganization, protein denaturation, and mild demyelination in the adjacent DREZ. Multiparametric MRI provides a sensitive, noninvasive approach for characterizing region‐specific changes and holds promise for monitoring injury progression and evaluating therapeutic interventions.

## Funding

This work was supported by National Institute of Neurological Disorders and Stroke (NS092961).

## Data Availability

The data that support the findings of this study are available from the corresponding author upon reasonable request.
